# Our Advertisers

**Published:** 1888-01

**Authors:** 


### OUR ADVERTISERS.

Next to an appreciative and "good pay" patient, there is no class of people whom we welcome with greater pleasure in our sanctum than the representatives of any of our numerous advertisers. Our list of advertisers is one we are justly proud of. Reliable in their statements, as well as in their dealings, always engaged in the pursuit of excellence and superiority on all former achievements, prompt in their remittances to the medical press when due, they are, as a class, earnest, patriotic, indomitable workers, and the medical profession is in a great measure indebted to them for many of the methods which have of late years revolutionized the course of practice. We therefore reiterate the assertion, that we always greet such men with unfeigned gratification and readiness to express those sentiments, "*ore et scripto.*" Parke, Davis & Co. are a monumental firm, a Colossus, whose gigantic finger has pointed out to the medical world many a reform, many a remedy, and, we might add, many a startling revelation! Who doubts now that Kohler's Cocaine is but the evolved product of the Erytoxylon Coca, introduced, vaunted, and heralded for years in the medical press of both hemispheres by this energetic and enterprising firm?

The laboratory of Detroit gave the medical student of Vienna food for reflection. His brain was thus fecundated and brought forth the cocaine! This is but one out of the many results obtained by and gained to the science and art of medicine, thanks to the toilsome researches of Parke, Davis & Co. Can we ignore such men? No! Neither can the finger and pen of envy. That they should have enemies and invidious rivals amongst druggists and manufacturing chemists, is but natural, and more easily imagined than described. But so long as Parke, Davis & Co. will abide the rules they have adopted from the incipiency of their firm, respecting their relation to the medical profession and medical press, just so long will they be honored and sustained by both, and the croakings of enmity and rivalry will prove but "ephemeral squibbings," when applied to this staunch and reliable house.

